# Plant Life with and without Oxygen: A Metabolomics Approach

**DOI:** 10.3390/ijms242216222

**Published:** 2023-11-12

**Authors:** Vladislav V. Yemelyanov, Roman K. Puzanskiy, Maria F. Shishova

**Affiliations:** 1 Department of Genetics and Biotechnology, Faculty of Biology, St. Petersburg State University, 199034 St. Petersburg, Russia; 2 Department of Plant Physiology and Biochemistry, Faculty of Biology, St. Petersburg State University, 199034 St. Petersburg, Russia; puzansky@yandex.ru (R.K.P.); mshishova@mail.ru (M.F.S.); 3 Laboratory of Analytical Phytochemistry, Komarov Botanical Institute of the Russian Academy of Sciences, 197376 St. Petersburg, Russia

**Keywords:** hypoxia, waterlogging, flooding, submergence, anoxia, desubmergence, reoxygenation, metabolomics, adaptation, tolerance

## Abstract

Oxygen deficiency is an environmental challenge which affects plant growth, the development and distribution in land and aquatic ecosystems, as well as crop yield losses worldwide. The capacity to exist in the conditions of deficiency or the complete lack of oxygen depends on a number of anatomic, developmental and molecular adaptations. The lack of molecular oxygen leads to an inhibition of aerobic respiration, which causes energy starvation and the acceleration of glycolysis passing into fermentations. We focus on systemic metabolic alterations revealed with the different approaches of metabolomics. Oxygen deprivation stimulates the accumulation of glucose, pyruvate and lactate, indicating the acceleration of the sugar metabolism, glycolysis and lactic fermentation, respectively. Among the Krebs-cycle metabolites, only the succinate level increases. Amino acids related to glycolysis, including the phosphoglycerate family (Ser and Gly), shikimate family (Phe, Tyr and Trp) and pyruvate family (Ala, Leu and Val), are greatly elevated. Members of the Asp family (Asn, Lys, Met, Thr and Ile), as well as the Glu family (Glu, Pro, Arg and GABA), accumulate as well. These metabolites are important members of the metabolic signature of oxygen deficiency in plants, linking glycolysis with an altered Krebs cycle and allowing alternative pathways of NAD(P)H reoxidation to avoid the excessive accumulation of toxic fermentation products (lactate, acetaldehyde, ethanol). Reoxygenation induces the downregulation of the levels of major anaerobically induced metabolites, including lactate, succinate and amino acids, especially members of the pyruvate family (Ala, Leu and Val), Tyr and Glu family (GABA and Glu) and Asp family (Asn, Met, Thr and Ile). The metabolic profiles during native and environmental hypoxia are rather similar, consisting in the accumulation of fermentation products, succinate, fumarate and amino acids, particularly Ala, Gly and GABA. The most intriguing fact is that metabolic alterations during oxidative stress are very much similar, with plant response to oxygen deprivation but not to reoxygenation.

## 1. Introduction

Oxygen deficiency is among the stress factors which cause severe harm to plants. Local intensive rainfall, poor drainage, snow and ice covering, followed by thawing, as well as global warming and the subsequent elevation in the sea level and precipitation are the reasons for partial or complete flooding [1,2,3]. The waterlogging impairs the gas (including oxygen) circulation in the rhizosphere, leading to limited oxygen availability in roots. Complete submergence of a plant brings about total oxygen depletion. The low diffusion of molecular oxygen first results in the development of gradual hypoxia (from 15 to 1% of O_2_) and anoxia (0% O_2_) with the lapse of time. Oxygen deficiency affects plant growth, development and distribution in terrestrial and aquatic ecosystems, as well as crop yield losses worldwide [2,4,5].

**Figure 1:**
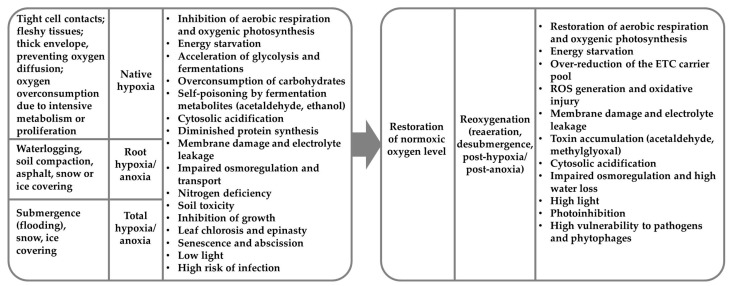
Impact of oxygen deficiency/reoxygenation stress of different origins on plants. Based on [1,2,5,6,7,8,9,10,11,12,13,14].

The capacity to exist in the conditions of deficiency or the complete lack of oxygen depends on a number of anatomic, developmental and molecular adaptations. The inhibition of axial organ growth, leaf chlorosis and epinasty, the abscission of leaves, flowers and fruits, premature senescence, finally ending with plant death, occurs in the majority of susceptible wild and cultivated plants under oxygen limitation (Figure 1) [2,6,7,8]. In such adverse conditions, aerobic respiration and photosynthesis are blocked, which ultimately causes energy starvation. The imposition of anoxia depletes the level of cellular ATP within a few minutes [6]. Glycolysis becomes the only way to synthesize ATP. The reoxidation of NAD(P)H takes place during lactic and ethanolic fermentations, which are stimulated by low oxygen. Overconsumption of reserve sugars for glycolysis enhances energy starvation, particularly in hypoxia-susceptible species [2,7,8]. On the other hand, the stimulation of lactic fermentation and the lack of ATP to energize the transport of ATPases accounts for cytosol acidification [2,6,7]. Alcoholic fermentation produces a toxic intermediate (acetic aldehyde) and end products (ethanol) that, together with acidosis, self-poison the plant under oxygen deficiency [2,7,8]. In natural habitats, flooding leads to a low soil redox potential and the production of reduced substances, including Fe^2+^, Mn^2+^, H_2_S and NO_2_^–^, along with the products of the carbon metabolism, such as methane, ethane, ethylene, acetylene, acetic and butyric acid, etc. [2,6]. Moreover, the re-establishment of normoxic conditions triggers the oxidation of these substances and the synthesis of reactive oxygen, nitrogen, carbonyl and sulfur species, resulting in post-anoxic injury [1,2,3,4,5,6,7,9,10].

However, there is a group of hydrophytic plants that is capable of growing under the conditions of submergence, and even complete anoxia. Wetland plants survive the oxygen deficiency by the use of two adaptation strategies. The first is the strategy of escaping the oxygen-deficient environment (low-oxygen-escape syndrome, LOES). Submergence accelerates the growth of LOES in plant axial organs, and stimulates the formation of adventitious roots and aerenchymatous tissue [1,2,7,8,9,15]. As a result, the shoots begin the transport of air into the flooded parts of the plant. Flooding also enhances the formation of aerial films on the nonwettable shoot surface, which facilitates gas flow between the air and the submerged part of the plant [1,16] and stimulates underwater-shoot photosynthesis [17,18,19]. LOES is strictly regulated by ethylene and gibberellins by the stimulation of intercalary meristem activity and internode/petiole growth. The energy required for accelerated growth comes from the GA-stimulated starch breakdown [1,2,5,7,8,9,15,20,21]. LOES is typical for most hydrophytes, including deepwater rice, *Rumex palustris*, *Rorippa amphibia*, etc. [5,22].

The second strategy is the quiescent strategy (low-oxygen-quiescence syndrome, LOQS). It consists mainly of metabolic adaptation and affects the mechanisms that prevent energy starvation, the toxicity of fermentation products and post-anoxic oxidative damage. LOQS is characterized by a decrease in the growth rate and leaf senescence, limited GA-dependent starch breakdown, the anaerobic metabolism and the conservation of metabolic energy, which is used predominantly for the synthesis of proteins involved in membrane transport, ROS protection and chaperone activity [1,9,23]. LOQS is common in *Sub1* rice varieties, *Arabidopsis thaliana*, *R. sylvestris*, *Oenathe aquatic*, etc. [5,22].

Both strategies lean on a metabolic background that allows tolerant plants to produce enough energy, maintain water and mineral uptake and even grow in oxygen-deficient environments (for LOES plants). Most of our knowledge on the plant hypoxic/anoxic metabolism has been obtained by the methods of conventional biochemistry, when the content of one or another metabolite, or group of metabolites, was studied in a targeted way. With such an approach, even significant changes in the level of other compounds that were not the original goal of the analysis might be outside the scope of the study. To solve these problems, “omics”-biology methods are currently employed. These permit the complex study of alterations in a pool of low-molecular-weight compounds, which can be considered as an integrative result of metabolic processes occurring at different levels of organization: in the cell–tissue–organ–body of the plant. Methods for the analysis of metabolite profiles based on chromatography first began to be used in medical clinical research as early as the 1960s–1970s. But only since the 1990s have methods of extraction, derivatization, chromatography and detection, as well as the following multivariate statistics analysis, been developed that are applicable to the study of plants. With the improvement of a systemic approach in biology, by analogy with the terms “genome” and “proteome”, in 1998, the term “metabolome” was proposed to refer to the entirety of all metabolites of a biological system [24,25]. Almost simultaneously, the term “metabolic profiling” appeared to denote methods allowing the analysis of a wide range of metabolites in a single sample. Since that time, the acceleration of research aimed to study the plant metabolome has been distinguished. This approach was in use for the phenotyping of varieties, populations and plant species. Metabolome analysis was employed to study processes, not only in model objects, but also in agricultural and wild plants. The advantages of metabolic profiling ensure investigations of complex alterations in the plant metabolome during plant development and the detection of the biochemical peculiarities of different plant organs/tissues under normal and modified environments.

Recent investigations are directed in the elucidation of metabolic variability under the effect of the both abiotic and biotic stress factors (desiccation, salinity, low and high temperature, pathogens, etc.). Unfortunately, the number of studies focused on the metabolic profiling of plants under hypoxia/anoxia is rather limited in comparison with those related to another type of stressor [26]. Nevertheless, the spectrum of available data indicates their specificity for oxygen deprivation affecting diverse species and plant organs. The obtained metabolomics dataset and further facility to apply statistical methods for its analysis enhance the biological interpretation of the revealed mechanisms (molecular networking, pathway analysis, biological modeling, etc.) of plant resistance to oxygen deficiency. It opens perspectives to highlight the metabolic steps crucial for biochemical adaptation during oxygen deprivation. This review focuses on common and specific metabolic adjustments of the primary metabolism appearing in tolerant and sensitive species as a feedback to oxygen shortage. We will consider a number of questions recently being addressed to biochemical alterations triggered throughout subsequent reaeration and their correspondence to processes changed under oxidative stress.

## 2. Specificity of Plant Metabolomic Profiles during Oxygen Lack

In the last two decades, the specificity of alterations occurring at the level of individual metabolites or its groups under oxygen deprivation has been detected with different technological platforms: gas- and high-performance liquid chromatography coupled with mass spectrometry, as well as NMR detection (Table S1). Further nontargeted or targeted quantitative profiling has resulted in a wide spectrum of data. The hypoxic/anoxic metabolomes of *A. thaliana* [27], *Lotus japonicus* [28], rice [29,30], soybean [31,32], wheat [33,34] as well as wetland species like *Zostera marina* [35], *Potamogeton anguillanus* [36], *Epilobium hirsutum* and *E. palustre* [37] have been studied (Table S1). 

Principal component analysis (PCA) is commonly applied in untargeted metabolomics to reveal the differences in the metabolic dataset. For example, the comparison of the differences between metabolomes of two *Triticum aestivum* cultivars under flooding conditions indicated that PC1 explained 38.5% of the variance separating the days of submergence, while PC2 and PC3 explained 25.3% and 7.21% of the variance, respectively [33]. PC3 indicated the separation between the two cultivars. The comparison of the metabolic profiles of two soybean cultivars profiles showed PC1 and PC2 values of 36.6 and 19.5%, respectively. The different metabolic responses obtained differed in terms of the tolerance to flooding stress, not only between the tested cultivars, but also for the roots and leaves [32]. The provided short list of examples indicates the high accuracy of the applied approach in allowing for the recognition of the importance of plant sensitivity to oxygen deprivation, tissue/organ specificity as well as the duration of the application and the intensity of the stressor. After the PCA elucidates the main trend of the differences, supervised methods, such as OPLS-DA, may be employed. The main purpose is to reveal the marker metabolites of the most responsive metabolic pathways providing tolerance to oxygen deprivation. As a result, such metabolic processes as glycolysis, the Krebs cycle and the amino acid metabolism were supposed to be involved in the adjustments of the plant metabolism to conditions of oxygen limitation.

The abovementioned list of species for which metabolic changes in the primary metabolism have been characterized indicates that the tolerance of the studied plants varies over a wide range. In this regard, during the comparative analysis of the data in this review, we divided them into several groups. First, we examined the changes occurring in the model plant, Arabidopsis; then, in another model object, rice, characterized by a high tolerance to oxygen deficiency. Next, we continued by considering the metabolic profiles of a number of other adapted wetland species, including those which tolerate salinity. The consideration is completed by a group of woody plants, cereals and legumes that differ from rice in being much less tolerant to hypoxia/anoxia (especially wheat and barley).

### 2.1. The Level of Glucose, Fructose and Other Carbohydrates

One of the most severe injuries during oxygen deficiency is the intensive lack of energy. The intensification of glycolysis for ATP synthesis and to regenerate NAD^+^ through alcoholic fermentation is the most important metabolic mechanism developed during the transition of the metabolism from aerobic to anaerobic. Those processes require alteration in carbohydrate consumption [38]. Thus, the alteration in the carbohydrate profile is an important trace of the metabolic adaptation to oxygen lack.

The model object *A. thaliana* is known to be middle-tolerant to oxygen deficiency. One of the early metabolomics studies showed that, in *A. thaliana* seedlings, oxygen deprivation triggered the elevation in glucose, fructose and other monosaccharide levels (all the experimental conditions of the discussed investigations and alterations of the major metabolite levels are summarized in Table S1). The most significant alterations were detected in the roots in darkness under 4% oxygen [27]. Later, the absence of intensive changes in the level of fructose and glucose was detected after 2 h of anoxia in illuminated Arabidopsis seedlings grown in the medium supplied with 1% sucrose [39]. Meanwhile, another set of experiments (16 h of hypoxia in the darkness) led to the accumulation of fructose and mannose, but not glucose [40]. Alteration in Arabidopsis sensitivity due to different genomic approaches was found to be important for the carbohydrate profile. The expression of the N-end-rule-insensitive *Δ13RAP2.12* of the ERF-VII factor RAP2.12 led to the slight increase in sucrose [40]. The cellular levels of sucrose and fructose were not changed under anoxia, and the dynamics of the carbohydrates were quite similar in wild-type Arabidopsis seedlings and mutants defective in glutamate dehydrogenase, *gdh1gdh2* [41]. The slight difference was revealed for glucose, whose content increased in the mutant. Various *A. thaliana* genotypes, differing by the presence/absence of the functional allene oxide synthase (AOS) and hydroperoxide lyase (HPL), showed the accumulation of glucose, *myo*-inositol, sucrose and raffinose-family oligosaccharides (raffinose, stachyose, verbascose) during waterlogging [42]. These alterations were most notable in the most hypoxia-intolerant *aos hpl* genotype.

Sugar profiles were investigated in a number of species known to be tolerant to oxygen deprivation. Anoxic treatment led to a decrease in the hexose level in rice coleoptiles, while sucrose accumulated, particularly after 6 d of oxygen lack [43]. Submergence (3 days) of rice seedlings (*O. sativa* L. ssp. *japonica*) of the cultivar M202 and its near isogenic, *Sub1A* introgression line, M202 (Sub1), had a strong impact on the reduction of the sucrose level in the leaves of both genotypes. Nevertheless, sucrose and glucose were significantly increased in M202 (Sub1) [44]. In another study, the comparison between weedy rice WR04-6 (hypoxia-tolerant) and QSZ rice (Qishanzhan, hypoxic-germination-sensitive (HGS) *O. sativa* L. ssp. *indica*) cultivars indicated hypoxia-induced sucrose accumulation in coleoptiles, which was more pronounced in WR [45]. The partial submergence of deepwater (NIL-12) and nondeepwater rice cultivars (Taichung 65 (T65)) showed the continuous increase in sucrose, fructose and glucose content upon the prolongation of oxygen deprivation [30]. Carbohydrate balance was completed with the decrement of the starch content in the leaves.

Now we will consider investigations provided on seagrasses, which are supposed to be even more tolerant to hypoxia. Oxygen deprivation of different intensities (hypoxia and anoxia) led to counter directional changes in the starch and soluble sugars in the shoots of pondweed *P. anguillanus* [36]. Quite different were the dynamics of glucose and fructose in eelgrass *Z. marina* [35]. Anoxia triggered a slight elevation in the levels of both sugars in the leaves, especially during the daytime, while in the roots, the fructose level significantly dropped and was not affected for glucose.

A specific experimental model concerns the seedlings of *Suaeda maritima* [46]. Submergence with sea water decreased the glucose and fructose in the leaves, and the intensity of that was consistent with the water salinity. The tendency in the roots was the opposite. Moreover, the level of trehalose increased. Hypoxia upraised the level of glucose and fructose in the roots, and fructose only in the leaves of *Beta vulgaris* seedlings with the additional application of salt stress [47]. These changes were accompanied by sucrose and trehalose elevation.

In the roots of young plants (3 months old) of flooding-tolerant gray poplar (*Populus × canescens*), one week of hypoxia increased the levels of glucose, fructose and sucrose [48]. These dynamics were accompanied with a decrement in the starch level. Only a minor decrease was recorded for sucrose in leaves, while other tested carbohydrate contents were stable. Waterlogging of the alpine plant *Rhododendron delavayi* for 30 days caused a significant accumulation of soluble sugars, including sucrose and glucose in leaves [49].

In the case of *T. aestivum*, the anoxia treatment triggered the decrement of soluble sugar levels in coleoptiles [43]. Prolonged submergence of two cultivars of *T. aestivum*, differing in their resistance (intolerant Frument and tolerant Jackson), led to decreases in the sucrose, fructose and glucose content in the shoots of both genotypes [33]. Another two wheat genotypes were tested for their responses to 1 day of anoxia at different temperatures [34]. The sugar profiles of the coleoptiles and the roots of both the more-tolerant Calingiri and less-tolerant Ducula cultivars decreased at all the tested temperatures during anoxia, but higher temperatures led to severe impacts. Only the fructose level was elevated in Ducula coleoptiles at 28 and 15 °C. Hypoxia (3–6 h) induced the rather fast depletion of sugars (sucrose, glucose and fructose) in the root tips of *Zea mays* [50]. Remarkably, the addition of exogenous glucose in media did not prevent this effect of oxygen deprivation.

In *L. japonicus* roots (which was shown to be more tolerant to prolonged waterlogging in the case of nodulation), a drop in both fructose and glucose was detected both in wild-type and transgenic plants (LbRNAi), characterized by the reduced activity of genes encoding leghemoglobin in the nodules [28]. A sharp decrease in starch was also revealed. Similar dynamics were determined in the nodules of the wild-type and transgenes. Twelve days of submergence of the soybean seedlings of two cultivars (BR4 and E45) led to the increase in sucrose but the decrement of the glucose level in the leaves [32]. In the case of *Medicago truncatula*, root flooding declined the level of raffinose, sucrose, hexoses and pentoses. The leaves, by contrast, were characterized by the increase in sugars and sugar derivatives [51]. The investigation of the effect of the O_2_ concentration (21, 2 and 0%) on sunflower (*Helianthus annuus*) showed that oxygen deficiency caused the increase in sucrose and fructose levels in the leaves, but that of glucose was decreased [52].

Thus, in the case of O_2_ limitation the levels of sugars alter in the different organs and tissues of plants which differ in their sensitivity to hypoxia/anoxia. Tolerant plants are characterized generally by the accumulation of low-molecular sugars, while intolerant ones by the decrease in soluble sugar levels. This carbohydrate recourse is usually directed in glycolysis. The activation of this process is closely linked with the accumulation of phosphorylated intermediates.

### 2.2. Intermediates of Glycolysis

Phosphorylated glycolytic intermediates glucose-6-phosphate and glycerol-3-phosphate showed a decrease in *A. thaliana* roots after a short (30 min) exposition at low O_2_ concentrations. Prolongation of the treatment at low-oxygen concentrations (48 h) caused the opposite response, as well as a decrease in the O_2_ concentration up to 4% [27]. After 16 h of hypoxia in the darkness, the leaves of wild-type *A. thaliana* without the application of external sugars showed no significant changes in the content of glucose-6-phosphate, fructose-6-phosphate, other hexose phosphates, glycerol-3-phosphate and even pyruvate, while the 35S::*Δ13RAP2.12* transgene was characterized by a higher content of glycolytic metabolites and their induction in response to hypoxia [40]. In *A. thaliana* seedlings (wild-type and *gdh1gdh2* mutants) after 4 h of anoxia, the levels of glucose-6-phosphate and fructose-6-phosphate sharply decreased [41]. *A. thaliana* genotypes with depressed oxylipin synthesis were characterized by significantly decreased UDP-glucose, hexose phosphates and increased trehalose-6-phosphate under soil waterlogging [42].

After 1 day of anoxia, there were no changes observed in the level of identified glycolytic intermediates in rice coleoptiles compared to the control, but after 6 days of anoxia, the levels of glucose-6-phosphate and fructose-6-phosphate increased, as well as 3-phosphoglycerate [43]. Glucose-6-phosphate and fructose-6-phosphate showed an upward trend in the leaves of T65 rice during the first hours of partial submergence under illumination; then, after 3 days, their levels strongly decreased. In the NIL-12 genotype, this was not observed, and the level of phosphates was relatively high [30]. Three days of flooding did not affect the levels of glucose-6-phosphate and 6-phosphogluconate in the leaves of rice seedlings (M202 and M202 (Sub1)), while the level of 3-phosphoglycerate significantly decreased [44].

Hypoxia caused the accumulation of phosphorylated intermediates of glycolysis (glucose-6-phosphate, fructose-6-phosphate and 3-phosphoglycerate) in sugar beet [47] as well as fructose-6-phosphate in gray poplar roots [48].

At the same time, 4 h of anoxia in wheat was already enough to cause a decrease in sugar phosphates. But after 6 days of exposure, their level was, on the contrary, elevated. At the same time, no changes in 3-phosphoglycerate were registered. Interestingly, both rice and wheat showed a decrease in glycerate levels in response to hypoxia, regardless of the time of application [43]. A rather complicated dynamic of sugar phosphates was detected in the different organs of *T. aestivum*, depending on the anoxia duration and experimental temperature. Coleoptiles of the seedlings grown at 15 °C did not show significant changes. The exception was the SARK variety, which showed a decrease in glucose-6-phosphate. In the roots at this temperature, anoxia caused a drop in the level of fructose-6-phosphate in two varieties (Carnamah and Calingiri), while the level of glucose-6-phosphate decreased in Ducula and increased in Calingiri. At 28 °C, the situation changed significantly: coleoptiles of the seedlings of three of five tested varieties (Spear, SARC and Ducula) showed a significant drop in the content of both phosphates, contrary to the Carnamah variety, which demonstrated an increase. In the roots at 28 °C, anoxia caused a decrease in the level of fructose-6-phosphate in two varieties (SARC and Ducula), while in none of the varieties was the level of glucose-6-phosphate affected. A study of the effect of hypoxia at 28 °C, 24 °C, 20 °C and 15 °C showed that, in general, an increase in temperature led to a greater decrease in the level of glucose-6-phosphate and fructose-6-phosphate, but there was no direct similarity with the alteration in the level of hexoses, as described above [34]. Twenty-four hours of hypoxia also caused the alteration of sugar phosphates in the leaves of *Hordeum vulgare*. Glucose-6-phosphate and fructose-6-phosphate increased under hypoxia in the wild-type, but in hemoglobin-overexpressing plants (HO), only glucose-6-phosphate was elevated. The level of 3-phosphoglycerate and pyruvate was significantly higher during hypoxia, particularly in HO plants [53]. In soybeans, within 3–6 h of hypoxia, the content of fructose-6-phosphate and pyruvate in the roots was not changed. The level of glucose-6-phosphate first showed an increase after 3 h of hypoxia, but the prolongation of the oxygen lack (6 h) triggered the drop below the control [31]. 

Literature data confirm that the common tendency is a decrease in phosphorylated glycolytic intermediates, especially in hypoxia-susceptible species.

### 2.3. Metabolites of Fermentation

Metabolically, ethanolic and lactic fermentations are a continuation of glycolysis due to the inability of oxidation by molecular oxygen in an anoxic environment. Basically, platforms used for metabolic profiling are not suited for ethanol detection. It is lost during sample extraction and preparation. Quite often, the accumulation of lactate is detected in metabolomes of different plant tissues under the oxygen lack.

Seven-day-old seedlings of *A. thaliana* after 2 h of hypoxia (with 1% sucrose in the media) under illumination showed a sharp increase in lactate levels in both the leaves and roots [39]. But, hypoxia in the darkness without sugars did not significantly affect the levels of ethanol and lactate in the leaves of *A. thaliana*. The overexpression of the *D13RAP2.12* construct significantly stimulated the accumulation of these fermentation products under normoxia and, especially, hypoxia [40]. Both the wild-type 7-day-old seedlings of *A. thaliana* and the *gdh1gdh2* mutant accumulated lactate during 4 h of anoxia [41], while the lack of oxygen during illumination did not cause an increase in the end products of fermentation, such as ethanol and lactate, in the detached leaves of *A. thaliana* and sunflower [52].

Three days of submergence did not significantly affect lactate levels in wild-type rice and the *SUB1A* mutant. However, *SUB1A* plants accumulated a larger pool of lactate [44]. The rice deepwater line NIL-12 and its background T65 differed in the elevation in lactate during flooding. In the first 12 h, lactate accumulation was less intensive, and intensified in the period of 24–72 h, but further on (168–288 h), the differences between lines weakened [30]. 

Anoxia, but not hypoxia, resulted in the elevation in lactate levels in the shoots of pondweed *P. anguillanus* [36]. In the leaves and roots of eelgrass *Z. marina*, the anoxic effect depended significantly on a stage of the diurnal cycle: during the light period, the lactate pool was stable, but increased dramatically in the darkness [35]. In the roots, but not in the leaves, of poplar, lactate and ethanol accumulated during the first hours of hypoxia, and then their levels decreased to the initial values [48].

During 5 days of flooding, lactate alteration in the roots and nodules of *L. japonicus* demonstrated a similar pattern—first, a sharp increase, and then a downgrade to the initial level. The silencing of three homologous genes encoding leghemoglobin in the nodules did not affect this pattern [28]. In soybean, within 3–6 h of hypoxia, the content of lactate in the roots increased. Under hypoxia, the redistribution of the C^13^ label from pyruvate to lactate sharply increased as a result of fermentation induction [31]. An increase in the level of acetate in the roots of *Glycine max* (an intolerant plant) was observed after several days (2–12) of flooding. Its level in the leaves slightly decreased after 12 days. Responses were the same for the sensitive variety BR4 and the moderately tolerant Embrapa 45 [32]. Twenty-four hours of hypoxia resulted in lactate accumulation in the leaves of the *H. vulgare* wild-type and hemoglobin-overexpressing (HO) plants [53].

Thus, regardless of the plant species and its sensitivity to a lack of oxygen, lactate accumulation is recorded under the lack of oxygen.

### 2.4. Metabolites of the Krebs Cycle

Along with carbohydrates, the oxygen deficiency affects the balance of organic acids due to the alterations of the reactions of the Krebs cycle. Succinate dehydrogenase activity requires the operation of the mitochondrial electron-transport chain and would be very much inhibited due to the limitation of molecular oxygen. The Krebs cycle splits up into two independent branches, starting from oxaloacetate and routing either to malate–fumarate–succinate (the reversal of the dicarboxylic part) or to 2-oxoglutarate (the oxidative tricarboxylic part) [2,28,31,44].

Oxygen deprivation led to some accumulation of Krebs-cycle intermediates: succinate, fumarate and citrate in the seedlings of *A. thaliana*. This trend was most pronounced at 4%, and to a lesser extent, at 1% O_2_ [27]. After 2 h of hypoxia under light conditions for Arabidopsis seedlings, the content of malate and 2-oxoglutarate was decreased in the roots, but did not significantly affect the shoots [39]. But, the intensification of stress conditions (16 h in the darkness without sugars in the incubation medium) did not lead to meaningful changes in the content of carboxylates in the leaves of wild-type *A. thaliana*. The basic levels of these metabolites were higher in the 35S::*Δ13RAP2.12* transgene [40]. After 4 h of anoxia in the wild-type *A. thaliana* seedlings, the levels of citrate, 2-oxoglutarate, fumarate and malate dropped, while the amount of succinate significantly increased. In the *gdh1gdh2* genotype, similar responses were observed, but often attenuated or delayed, and the 2-oxoglutarate level was higher [41]. Arabidopsis genotypes with impaired oxylipin biosynthesis accumulated higher amounts of organic acids related to the Krebs cycle (succinate and fumarate) and decreased the ascorbate under hypoxia [42].

The effect of short-term anoxia (4 h or 1 d) on the level of Krebs-cycle intermediates in rice coleoptiles was predominantly negative: the levels of aconitate, malate citrate, 2-oxoglutarate and isocitrate dropped, while the succinate content was elevated. Six days of anoxia, on the contrary, led to an increase in the levels of fumarate, aconitate and citrate, accompanied by a dramatic rise of succinate and the reduction of 2-oxoglutarate content [43]. Flooding (3 days of treatment) resulted in the intensive reduction of malate and succinate levels in both the M202 and M202(Sub1) rice seedlings. Citrate decrease was determined only in M202 [44]. Succinate showed an upward trend in the leaves of T65 rice during the first day of partial submergence under light, but with prolonged exposure, its level was greatly reduced. NIL-12 initially had a relatively high level of succinate and gradually decreased during flooding [30]. Hypoxia led to an increase in the pool of succinate in rice, and the effect was more pronounced in the tolerant variety WR in comparison with the sensitive QSZ. The level of malate responded to hypoxia in these cultivars in the opposite way: in WR—growth, in QSZ—a decrease [45].

In the case of the shoots of *P. anguillanus*, hypoxia did not affect the levels of citrate, isocitrate and malate. At the same time, during anoxia, their levels increased significantly. Fumarate accumulated under both hypoxia and anoxia, while the level of succinate did not change under these effects [36]. The succinate concentration showed an accumulation in both the roots and leaves of eelgrass *Z. marina* under anoxia. In addition, anoxia caused an elevation in 2-oxoglutarate in the leaves, and an increase in fumarate and a decrease in malate in the roots [35]. The leaves of the hydrophyte species of willowherbs *E. hirsutum* and *E. palustre* were characterized by the accumulation dicarboxylic acids of the Krebs cycle (fumarate and succinate) in their natural wetland environment [37].

Hypoxia had little effect on the level of carboxylates in seepweed shoots *S. maritima*, only causing a decrease in succinate in the background of high salinity. The accumulation of citrate, succinate and malate was shown in seepweed roots during flooding, especially with increasing water salinity [46]. Hypoxia caused an increase in the level of carboxylates of the Krebs cycle (succinate, fumarate, citrate, isocitrate and malate) in the roots of *B. vulgaris*. In the leaves, the reaction was reversed, with a decrease in citrate, succinate and fumarate [47]. The level of organic acids in the roots generally showed a tendency to rise, but the temporal patterns of the increase in the level of metabolites differed in gray poplars under hypoxia. Primarily, aconitate (5, 24 h) showed a significant increase; then, malate (24 h), succinate (24, 168 h) and 2-oxoglutarate (24, 168 h) joined. The last two metabolites increased in concentration after a week of exposure [44]. Thirty-day waterlogging led to the accumulation of malic, citric and linolenic acids in the leaves of *R. delavayi* [49].

In wheat coleoptiles, the effect of anoxia on the level of most Krebs-cycle metabolites was rather negative, regardless of the duration of exposure, but not for succinate, which was found to be accumulated [43]. The comparison of five cultivars of *T. aestivum* represented the negative consequences for carboxylic acids in the both coleoptiles and the roots of Spear, SARC and Ducula. It was more pronounced at higher temperatures. Alternatively, the level of succinate increased in the roots of four among the five tested cultivars at a higher temperature regime. At 15 °C, its accumulation was determined only in two cultivars (Calingiri and Carnamah), and in two (Spear and SARC), the dynamic was the opposite. A specific reaction was estimated for Spear, which was shown to accumulate *cis*-aconitate [34]. Reduced levels of citrate and malate were observed in the tips of the primary roots of *Z. mays* under hypoxia. In the case of succinate, an increased concentration was observed in the period of 3–6 h (the growing medium contained a high level of sucrose). After 24 h of hypoxia, the succinate concentration was already lower than during normoxia [50]. A unique case is that hypoxia had no effect at all on the level of the Krebs-cycle metabolites in *H. vulgare* [53].

Hypoxic treatment stimulated the accumulation of succinate and the depletion of malate in the roots and nitrogen-fixing nodules of both genotypes of *L. japonicus*, while the citrate level was unchanged and the 2-oxoglutarate one increased in the leghemoglobin knockdown only [28]. After several days of flooding of the *G. max* seedlings, a decrease in the fumarate and citrate contents in the leaves was observed in both the susceptible cultivar BR4 and the resistant E45. The malate concentration very slightly reduced in BR4 and slightly accumulated in E45. In the roots, the reaction was different. The level of fumarate also decreased in both varieties, while in E45, it was already lower, and the decrease during hypoxia was little, so that, after hypoxia, the content in both genotypes leveled off. The citrate content, on the contrary, increased to a greater extent in E45. The pool of malate decreased in BR4 and did not change in E45. Since the level of this metabolite was already low in E45, the final content in both varieties leveled off. The level of succinate in the roots increased sharply in both varieties [32]. In soybean during 3 and 6 h of hypoxia, the citrate and 2-oxoglutarate concentrations did not change in the roots, while the level of fumarate and malate slightly decreased after 6 and 3 h, respectively. The content of succinate was higher after both time points. During hypoxia, the ^13^C label from pyruvate showed a greater accumulation of succinate and less of malate and fumarate [31]. 

Thereby, the depletion of oxygen generally leads to a decrease in Krebs-cycle carboxylates, with the exception of fumarate and succinate, particularly in more resistant plants.

### 2.5. Amino Acid Metabolism

Another group of metabolites sensitive to oxygen limitation is nitrogen compounds. It is closely linked to the utilization of pyruvate and its further association with the metabolism of amino acids.

The incubation of seedlings of *A. thaliana* at oxygen deprivation changed the amino acid profile in the roots. The low-oxygen concentration (1%) after 0.5, 2 or 48 h of incubation caused the decrease in the amino acid content: Ala, Glu, GABA and Pro. Surprisingly, at 4% oxygen, there was a general trend towards an increase in the concentration of the most amino acids, but at 8% of O_2_, the amino acid levels were similar to the control [27]. The combination of two hours of hypoxia with illumination resulted in a significant decrease in Asp, Asn and Glu, while Ala and GABA increased both in the shoots and roots [39]. Hypoxia (16 h) in the darkness without sugars added in the medium showed no changes in the content of amino acids (including Ala, GABA, Glu, Gln and Pro) in the leaves of *A. thaliana* seedlings, both the wild-type and the transgenes with the overexpression of *RAP2.12*. However, *Δ13RAP2.12* overexpression led to a decrease in respiration and a shift in the representation of Krebs-cycle intermediates with the accumulation of a large number of amino acids, including Ala and GABA [40]. After 4 h of anoxia, in the whole seedlings of *A. thaliana* (wild-type and *gdh1gdh2*), the levels of Ala and GABA increased, while the levels of Asp and Glu decreased [41]. Wild-type plants accumulated more Ala and GABA than the mutants. Arabidopsis plants with inactivated oxylipin biosynthesis responded to waterlogging with the accumulation of Leu, Ser, Pro and Tyr, and consistent decreases in Ala, Asp, citrulline and Met [42].

An increased content of amino acids was also observed in rice coleoptiles during anoxia. This effect was most notable after 6 days of anoxia: an elevation in the Ala, Arg, GABA, Gly, Glu, Gln, Ile, Leu, Val, Ser, Pro, Thr, Trp, Tyr, etc., content was observed [43]. In response to 24 h of submergence, the rice leaves of both M202 and M202(Sub1)—the submergence-tolerant *Sub1a* introgression line—showed the accumulation of 11 amino acids, such as Ala, Arg, GABA, Glu, Gly, Met, Phe, Pro, Thr, Tyr and Val. Furthermore, Arg, Phe, Pro, Thr and Val predominated in M202 and Glu in M202(Sub1). The level of Asp, on the contrary, decreased [54]. After 3 days of oxygen deficiency, its levels, along with Ser, Asp and Asn, were significantly elevated in both genotypes. The overall amino acid content was higher in M202 (particularly Ala and Asn) [29,44]. There was no significant difference in the amount of GABA between both genotypes [44,54]. Ala, Ser, Leu, Ile, Tyr and Val are synthesized from glycolytic intermediates or pyruvate, and their abundance in M202 during submergence indicated a greater carbohydrate consumption via glycolysis in the genotype less tolerant to hypoxia. In the leaves of the deepwater rice variety (NIL-12), compared to the nondeepwater rice variety (T65), the concentration of amino acids was reduced both in the control and after 24 h of submergence. In the NIL-12 line, which had the T65 genetic background, the amino acid content was also reduced, even without flooding, but to a lesser extent. This effect practically disappeared under hypoxia. Long-term submergence (up to 288 h) resulted in a decrease in the level of amino acids both in T65 and NIL-12 [30]. The accumulation of six amino acids (Arg, Ile, Met, Phe, Pro, Tyr and Val) was detected during the germination of the hypoxia-tolerant WR04-6 rice variety under hypoxic stress. Exceptionally, only the content of Glu decreased. In the sensitive QSZ seedlings, the content of five amino acids (Gln, Ile, Phe, Pro and Tyr) decreased, and that of three amino acids (Arg, His and Val) accumulated in response to hypoxic stress [45].

Prolonged hypoxia and anoxia (3 weeks) led to an increase in the level of free amino acids in pondweed *P. anguillanus* shoots. Pyruvate derivatives (Ala and Val) were significantly accumulated during anoxia. Higher levels of Asp and Asn, as well as Arg, GABA, Glu and Pro, were observed during anoxia as well. Interestingly, none of the amino acids were decreased. Moreover, the stimulation of amino acid accumulation was more pronounced during hypoxia than anoxia [36]. The pool of only two amino acids (GABA and Ala) expanded in both the leaves and roots under anoxia in eelgrass *Z. marina*. On the contrary, in the leaves, a number of other amino acids demonstrated decrement: Asp, Gly, Glu, Gln, Pro, Ser, Thr and Tyr. Moreover, in general, the decrease was intensified in the darkness. In the roots, vice versa, several amino acids (Ala, Asp, Ser and Tyr) showed an increased level, which was most notable in the light period. Glu, Gln, Pro and Thr, as in the leaves, showed a decrease under anoxia [35]. A greater accumulation of amino acids (α- and β-Ala, Asn, GABA, Glu, Leu, pyroglutamate, Phe, Ser, Thr, Trp and Val) was the characteristic feature of the hydrophyte species *E. hirsutum* and *E. palustre* growing in the natural wetland habitat [37].

An increase in free amino acids under hypoxia in both the shoots and roots was determined in seepweed *S. maritima*. The effects depended on an additional stressor. An increase in salinity stimulated the accumulation of amino acids associated with osmoprotection both under normoxia and hypoxia. For example, the increase in Pro during hypoxia was more embossed in the plant organs at a high salinity. In general, the responsiveness in the roots was stronger. The levels of Ala, Asn, Arg, His, Ile, Leu, Lys, Phe, Trp and Val increased in the roots in response to hypoxia and high salinity. GABA elevation was detected in the roots only. In the shoots, saline hypoxia induced Arg, Asn, Gln, Ile, Leu, Phe, Trp, Tyr, Val and β-Ala elevation. The opposite dynamics were estimated for Asp, Glu and Ser [46]. Under hypoxia, the concentration of free amino acids (including GABA, but not Ala and Pro) increased both in the roots and in the leaves of *B. vulgaris*. The effect was more notable in the roots than in the leaves; in the mature leaves, compared to the young ones, it was enhanced by salinity [47]. Only a few amino acids were negatively associated with O_2_ levels in the sunflower leaves exposed to the light, including Ala. The levels of other amino acids, including Gln, Gly, Leu, Met, Phe, Ser, Thr, Trp, etc., increased with O_2_ availability [52]. The intensity and direction of the alterations as a response to hypoxia of different durations (up to 168 h) were different in the tested organs of the poplar *P. canescens*. For example, Gly and Ser accumulated in the roots starting from the first hours of submergence. In the leaves, their content was decreased by 168 h. The levels of Ala, Arg, Gly, GABA, His, homoserine, Lys, Tyr and Val increased in the roots but not in the leaves [48].

In the case of two *T. aestivum* cultivars subjected to flooding (16 days), the metabolome analysis showed that 12 of the 17 evaluated amino acids (Asn, Gln, Ile, Leu, Lys, Met, Phe, Pro, Thr, Trp, Tyr and Val) increased in the shoots during the first 12 days. GABA did not show significant alterations in this period. A difference was observed in the pattern of amino acid accumulation between less-intolerant (Jackson) and more-intolerant (Frument) cultivars. Twelve days of submergence application resulted in a higher accumulation of amino acids in Frument compared with that in Jackson. Prolongation of hypoxia led to a gradual decrease in the amino acid level in Frument and an increase in Jackson (Leu, Lys, Met, Phe, Pro, Thr, Trp, Tyr and Val). Actually, the decrement in the amino acid pool by day 16 of the stressor action was found to be a common reaction, which was supposed to be explained by the general damage to plants [33]. Interestingly, intolerant cultivars accumulated higher GABA amounts, and tolerant cultivars accumulated Pro. The comparative analysis of the amino acid profiles in the seedlings (coleoptiles and roots) of five *T. aestivum* genotypes under anoxia, combined with different growing temperatures (15–28 °C), did not highlight the leading importance of any of the factors [34]. Under anoxia in the coleoptile of different wheat cultivars, a large number of amino acids showed an increase at 15 °C in comparison to that at 28 °C. The most characteristic changes were registered for Pro. A pronounced downward trend was shown for Asp, Glu, Gln and pyroglutamate. A significant accumulation of GABA was specific for all cultivars during the anoxic treatment. Similar drifts were observed in the roots [34]. The tolerant cultivar Calingiri was characterized by higher levels of amino acids, particularly GABA, Gly, β-Ala and Pro [34,43]. In *H. vulgare* seedlings (wild-type and the hemoglobin-overexpressing (HO) genotype), 24 h of hypoxia resulted in complicated alterations in the amino acid profile. Half of the amino acids (Asp, Glu, Gln, Ile, Leu, Lys, Ser and Thr) decreased, but another half (Ala, Gly, His, Phe, Pro and Tyr) increased. Only Pro and His showed significant differences between the two genotypes: their levels were higher in the wild-type. Both genotypes demonstrated the accumulation of GABA in the case of oxygen deficiency [53].

In *L. japonicus*, the effect of waterlogging on amino acids was similar in the roots and nitrogen-fixing nodules. In the last ones, the pool of soluble amino acids (Ala, GABA, Glu, Gly, Ser, Tyr and Val) increased more intensively due to the lack of oxygen. Silencing of leghemoglobin genes (the LbRNAi line) affected the balance of amino acids. In the roots of transgenic plants, the level of free amino acids was significantly lower than in the wild-type, and only Ala and Glu increased during flooding [28]. Shorter periods of hypoxia (3–6 h) elevated the content of Ala, Glu, GABA, Leu, Phe, Ser, Pro and Val in the roots of soybeans plants, but reduced it for Asp and Asn. Hypoxia induced the redistribution of the C^13^ label from pyruvate to Ala, GABA and Glu; to lesser degree, there was redistribution to Asp [31]. Several (2–12) days of flooding showed a shortage of the pool of Ala and GABA in the leaves of *G. max* plants. In the roots, the reaction was the opposite. Responses were similar for the sensitive cultivar BR4 and the moderately resistant Embrapa 45 [32]. In the leaves of *M. truncatula*, the levels of Ala, Gln, Gly, Lys, Phe, Trp and Tyr were increased under oxygen deficiency. But, the amplification of the Arg and Glu content was determined only in the first 2 weeks of flooding; by 3–4 weeks, they reduced to the control level. β-Ala and Asn showed a slight downward trend. In the roots, the boosting effect of flooding on the accumulation of amino acids was more pronounced. There were no amino acids in which the level was weakening. Flooding for 3 weeks (but not 1 week) resulted in a significant representation of Ala, Gly and Pro in the shoot phloem-sap-exudate levels. At the same time, the levels of many other amino acids (including GABA) did not change significantly [41]. 

Therefore, the accumulation of amino acids associated with the Glu metabolism and GABA shunt (Glu, GABA, Arg and Pro), as well as derived from the metabolites of glycolysis (Ala, Gly, Val, Ser, Phe and Tyr), was detected during oxygen deprivation in the majority of plant species regardless of their resistance to waterlogging/submergence.

## 3. Native Hypoxia and Metabolomics

Besides the extensively studied stress-induced environmental hypoxia, there are several developmental endogenic processes found to be characterized by the lack of oxygen in plant tissues (Figure 1) [11,12,55]. The intensive proliferation in the meristem is one of such native hypoxia cases. The first data on the possible role of hypoxia in stem-cell development were obtained with animal stem cells [56]. The reason for hypoxia was supposed to be an increasing O_2_ demand during rapid cell proliferation and a low-diffusion rate of O_2_. Later on, a similar lack of oxygen was determined in the lateral root primordia and shoot apical meristem [57,58]; in the bud meristem, covered with lignified bracts, thus preventing oxygen diffusion [59]; in the pathogen-induced galls [60]; at the site of *Botrytis cinerea* infection [61]. Unfortunately, we were not able to find data discovering metabolic alterations during all the listed internal hypoxia cases due to increasing oxygen consumption and/or impaired diffusion. Further development of metabolite-detection techniques is required to provide proper metabolic profiling, including single-cell analysis based on newly developed types of biosensors and/or mass spectrometry [62,63]. Nevertheless, the already-fulfilled single-cell transcriptomic analysis revealed the activation of the expression of genes involved in ethylene, ROS, ABA and hypoxia pathways during adventitious root organogenesis [64].

Another developmental process during which plant cells undergo hypoxia is germination. Respiratory activity following imbibition and the blockage of gas exchange due to the impermeable seed coat resulted in a decrease in the oxygen content, even in normally aerated soil [65]. Such conditions in seeds are followed by the suppression of aerobic respiration and the further activation of ethanolic fermentation. But, the time period of hypoxia during germination might be rather short. A number of metabolite profiles have already been applied to germination events in Arabidopsis, barley, maize and tomato [66,67,68]. Accumulated data indicate the intensive metabolic rearrangement, which is species- and genotype-dependent. Metabolic shifts were found in carbohydrate, nitrogen and the lipid metabolism. The early stages of tomato seed germination (6 h) were characterized by an intensive drop of sugar levels, but not of sugar phosphates (glucose-6-phosphate and glycerol-3-phosphate); an elevation in citrate and fumarate, but not of shikimate and succinate; an enhancement in amino acids associated with the Asp family (Lys, Thr, Met and Ile) as well as number of nonprotein amino acids (GABA) [67]. Similar data were obtained with rice seed aerobic germination, which has been analyzed for a longer time period. The authors compared the metabolomes of seeds at different time points, mainly in the early germination stages (0, 3, 6, 9, 12, 24, 36 and 48 h after imbibition), and provided two varieties with different speeds of germination [69]. Significant correlation with the germination rate was shown for the amino acids Val and Leu and their derivatives, including polyamines, as well as for flavonoids and high numbers of lipids, mostly represented by free acids. The main conclusion is that there are two metabolic modules that are critical for rice-seed germination. One is the Orn–Asn–polyamine module, and the other is the shikimate–Tyr–tryptamine–Phe–flavonoid module [69]. It was supposed that both modules cover the necessity of antioxidants. In the frame of the discussed subject, we may hypothesize that, at the early stages of hypoxia-tolerant rice seeds, the demand in antioxidants is programmed. 

Moreover, the intensity of the anaerobic metabolism was found to be different in cereals varying in the sensitivity to oxygen deprivation. The comparison of the embryos of seeds germinating at normoxia revealed that the ADH activity was higher in rice than in barley, and it increased continuously [70]. Very intriguing data were obtained with the intensification of the lack of O_2_, when the rice seeds were germinated under anoxia. A total of 10 metabolites were associated with aerobic germination, and 13 metabolites were defined as anaerobic responders [71]. Significant changes in the metabolite profile were detected already after 3 h of the absence of O_2_. The differences between the normoxic and anoxic variants were continuously increased after 12 h (20% of metabolites) and even 48 h (40%). Aerobic germination brought about the accumulation of sugars (arabinose and trehalose) and organic acids (citrate, gluconate, 2-oxoglutarate and shikimate). Anoxic conditions caused a decrease in the level of metabolites involved in the tricarboxylic part of the Krebs cycle: citrate, isocitrate and 2-oxoglutarate, but led to the accumulation of dicarboxylates: fumarate and succinate, the same as was discussed earlier for the plant hypoxic/anoxic metabolome. The elevation in succinate was supposed to be due to the inhibition of respiration and/or due to the linkage with the GABA metabolism. Germination under anoxia led to a shortage of shikimate, but a number of aromatic metabolites, such as the amino acids Phe and Tyr, phenylpropanoids, including monolignols, and flavonoids were found to be increased [69]. A limited number of investigations have covered all aspects of metabolic adaptations to the native hypoxia, and even the external intensification of this stress-factor ability to shift the metabolism of germinated seeds; further research is needed.

Recently, metabolic acclimations to developmental hypoxia were discussed for some plant storage tissues or organs, such as fruits [70]. Such oxygen deprivation is considered to be regulated and essential for maturation. The reasons for fruit hypoxia include the fruit skin, which is supposed to be a serious barrier for gas diffusion, the low porosity of fruit tissues and intensive seed respiration. Nevertheless, a low oxygen concentration is considered to delay the ripening and senescence of some fruits. Thereby, fruit adaptation to the native hypoxia acquires additional importance. A metabolomic approach was used to distinguish biochemical alterations on fruit ripening in different plant species, but no special attention was paid to the interrelation of the oxygen content and metabolite profile.

The most detailed metabolic profiling of tissue-specific hypoxia under normal air oxygen concentrations was provided with melon fruit [71,72,73]. The microsensor technique revealed that hypoxia was gradually established during fruit development and was increasing toward the melon fruit center, but there was no strong oxygen gradient between the outer and the inner mesocarp. Nevertheless, significant metabolic rearrangements occurred. PCA was performed with the averaged data of the estimated demands and the concentration of oxygen, as well as eighteen metabolites from the central metabolism in the five radial sections of the mesocarp. The PCA analysis showed that the first principal component (40% of the total variance) separated the developmental stages, whereas PC2 (32%) separated the studied sectors. In the course of fruit growth, the sucrose level was very low, while the glucose and fructose levels were the opposite. During ripening, the sucrose concentration elevated significantly, but the glucose gradient disappeared, whereas the fructose gradient was inverted. Pyruvate, fumarate and succinate showed a similar pattern. It increased during fruit maturity and the gradient from the aerobic periphery to the hypoxic center of the fruit was determined. On the contrary, the level of malate and citrate was low and, even during ripening, remained rather stable. Only at the stage of over-ripening did citrate show accumulation in the peripheral sector. Amino acids (Ala, Gln, Glu, Asp and GABA), including aromatic amino ones (Phe, Tyr and Trp), were accumulated in the melon fruit, and a slight gradient was observed from the periphery to the center of the fruit. Besides that, the accumulation did not correlate with protein degradation [73].

Several investigations used a metabolic approach to test changes in metabolic network and its shift during strawberry fruit ripening [74,75]. The levels of free amino acids decreased gradually before the red-ripening stage, but increased significantly in the over-ripening stage [74]. Among the increased ones were Glu, Met, Thr, Ala, Val, Gly, Phe and Tyr. Decreased levels were detected for organic acids: citrate, oxaloacetate and malate. Sugars and their derivatives showed a complex trend, mostly decreasing (fructose, galactose, *myo*-inositol-1-phosphate, *myo*-inositol and glycerol-3-phosphate). The authors did not associate the alterations with a possible hypoxia effect, but concluded that the amino acid metabolism is central to fruit development. It is necessary to note that strawberry fruits have low-porous tissues [70], and during the transition from the full-ripening to the over-ripening stage of fleshy fruit, the gas-filled intercellular spaces are blocked with water, and this might enhance hypoxia [76].

A complex investigation of the metabolic fluxes and enzyme activities in the tomato fruit pericarp included the detection of major metabolites, such as carbohydrates (glucose, fructose, sucrose and starch), organic acids (citrate and malate) and amino acids, as well as the activities of 36 enzymes involved in the carbohydrate metabolism, glycolysis and the metabolism of organic acids measured at nine stages of development [77]. The conclusion was that the metabolite profile was more responsive to environmental changes and that enzymes can control both metabolite concentrations and fluxes during ripening. Further modeling of the metabolite fluxes during tomato fruit maturation described the central metabolism over three phases of fruit development [78]. The flux profiles were classified into three mostly nonlinear patterns. Soluble sugars (glucose and fructose) slightly increased at the red-fruit stage. The regress was observed for sucrose and starch accumulation. Intensive accumulation for free amino acids and citrate was revealed at the final ripening of the tomato fruit. These results were also not compared with the possible effects of internal hypoxia establishment.

In large bulky fruits, like pears and apples, the lowest oxygen concentrations are found in the fruit core [70,79,80]. Unfortunately, metabolomics studies of apple fruit ripening are very few and do not consider the problems of native hypoxia. The levels of most of the sugars (fructose, sucrose, glucose, arabinose and trehalose) were increased, while organic acid, including Krebs-cycle intermediates, showed a distinct decreasing trend at the full-ripening stage. The levels of free amino acids (Asn, Thr, Cys, Gln, Lys, Asp, Glu, His and Ile) were increased as well [81].

Low-oxygen conditions are commonly used as a technological approach for controlled-atmosphere storage toward a better maintenance of commercial apple fruit quality during storage. Intensive metabolic rearrangements occurred during hypoxia in apples [82]. The metabolic profile of the apple cortex showed the hypoxia-induced accumulation of Ala, Thr Val, Met, Ser, Phe, GABA and ethanol [82,83]. A decrease was detected for Asn, Asp and uridine levels, and very slight one for some sugars (sucrose and galactose). The conclusion was that the reactions of apples to changes in the oxygen concentration are characterized by selective fine-tuned reconfigurations of the C and N metabolism.

A similar study of the metabolic profiles under external hypoxia in a controlled atmosphere was provided with the pear fruit [84]. The observations were, in the main, the same. The gradual development of oxygen deprivation in fruit tissues (5–2–1–0.5–0.2 kPa) had a negative effect on the content of sugars (sucrose, glucose and fructose) and led to the temporal accumulation of sugar phosphates, citrate, 2-oxoglutarate and malate. The almost anoxic condition of 0.2 kPa was marked by an increase in lactate, succinate, fumarate, Ala, Gly and Val.

In general, scanty literature data indicate a commonality of metabolic profiles during native and environmental hypoxia, consisting in the accumulation of fermentation products, succinate, fumarate and amino acids, particularly Ala, Gly and GABA.

## 4. Plant Metabolomic Profiles during Reoxygenation

Regardless of where and for what reasons the lack of oxygen appears, plant organisms in most cases return to normal aeration in the course of time. This phenomenon is easy to observe in the case of the both aquatic and terrestrial plants, which inhabit different ecosystems in flood-prone environments. A number of developed adaptations are responsible for the plant organism’s survival even during a long period of oxygen deprivation. Further desubmergence implies reoxygenation, and thus the restoration of such integrative processes as aerobic respiration and photosynthesis (Figure 1). It means the necessity of the rapid reprogramming of the plant biology to renewed conditions. However, peculiarities of plant survival after the transition to normoxia are still fragmented because the interest in this field developed much later than that concerning hypoxia/anoxia [85,86,87]. Surprisingly, the return to normal oxygenation puts plants under pressure from quite a wide range of stressors [9,10,88]. Under post-hypoxic/-anoxic conditions, nonenzymatic and enzymatic ROS scavenging is induced [10,89,90]. ROS accumulation and shifts in hormone balance are closely related to the acceleration of senescence, which are especially well-documented for leaves [50,91,92]. Besides the necessity to overcome oxidative stress, plants experience limitations in nutrition [93] and water supply [94]. The post-flooding period comes hand-in-hand with the elevation in immunity to insect attacks and pathogenic infections due to the different mechanisms, including the accumulation of so-called defense phytohormones [9,88].

Taken together, the accumulated data indicate a necessity of different mechanisms to overcome oxygen-deprivation consequences. In previous sections of our review, we focused on the role of metabolic alteration in the resistance to the submergence of various species. The most intriguing were the shifts in the metabolic profiles of carbohydrates and nitrogen- containing metabolites. Common limitations in the energy supply and the accumulation of toxic compounds characterized the period of O_2_ lack. The further oxygen-induced restoration of respiration and photosynthesis provided an expectation of the special importance of those groups. It was demonstrated that the limitation of plant survival during submergence might be caused less by the concentration of soluble sugars and starch and/or its faster depletion in susceptible species [95,96]. A very interesting investigation that compared carbohydrate levels was provided with three species: flood-tolerant *Polygonum hydropiper* and flood-sensitive *Phalaris arundinacea* and *Carex argyi* [97]. The results revealed that, during the submergence recovery period, carbohydrate levels increased more rapidly in more tolerant species. Similarly, in extremely tolerant *Distylium chinense* (seedlings are able to survive 150 days of flooding), the soluble sugars increased in the leaves and roots during the desubmergence [98]. For a short period of recovery, the tested carbohydrates in the leaves exceeded that in the control seedlings, but rather quickly, both were equalized. The content of nitrogen-containing metabolites during desubmergence was also investigated. Special attention was paid to Ala, characterized with an intensive accumulation even among other amino acids under oxygen deficiency. Nevertheless, in the roots of noninoculated soybean (*G. max*) plants, the restoration of aeration (reoxygenation) triggered the fast depletion of Ala to control the normoxic level [99].

The mentioned results were obtained with the methods of conventional biochemistry before the wide application of metabolomics and do not always provide a full scenario of the metabolic rearrangement. Up to now, we found only very few of such investigations based on the metabolomics approach.

The analysis of the post-anoxic metabolome of middle-tolerant *A. thaliana* seedlings after 6 h of reoxygenation, preceded by 4 h of anoxia, first revealed the temporal accumulation of sucrose, followed by the decrease to control the normoxic level (see Table S1) [37]. This is the second part of the study of the *Arabidopsis* metabolome provided with GC-TOF-MS that was discussed already in Section 2. The fructose content was not affected by the oxygen availability, and the glucose one was depleted. The differences between the wild-type plants and the mutants of *gdh1gdh2* defective in glutamate dehydrogenase included a decrease in sucrose levels under reoxygenation [37]. Exhausted by anoxia, pools of sugar phosphates were filled in to normoxic levels in the wild-type plants and exceeded those in the mutants upon the onset of post-anoxia. Pyruvate accumulated in both plants, but in the wild-type, it was faster than in the *gdh1gdh2* mutants. A significant drop in the lactate level due to reaeration was observed in both plants. Anoxia-depressed levels of carboxylates of the Krebs cycle either were kept unchanged (fumarate) or increased by reoxygenation (citrate and malate), indicating the stimulation of the pathway. These alterations were the most notable in the wild-type genotype. The 2-oxoglutarate level was steady in the wild-type plants and decreased in the mutants. Significantly elevated during oxygen lack, the level of succinate decreased to the level of the normoxic control, as well as those of GABA and Ala, but the last two remained above the control during reoxygenation. Wild-type plants contained more GABA and glutamate dehydrogenase, while mutants accumulated more Ala in post-anoxic conditions. The Glu and Asp content depleted by the oxygen shortage increased in both genotypes during reaeration, with higher accumulation in the mutants [37].

Metabolome profiling with the GC-MS approach detected complex alterations in *Arabidopsis* seedlings during 24 h of anoxia and a further 24 h of reoxygenation [87]. About 60% of the detected compounds were affected by anoxia, and only 26% were changed during further reaeration. The restoration of the O_2_ availability maintained high levels of sugars (glucose and fructose), as well as the inositol one created by anoxia. Even after 24 h of post-anoxia, it was higher than in the control. The content of gluconate and xylose increased as well. Phosphorylated substances were not really the focus of this investigation, but the intensity of fermentation could be evaluated through the lactate concentration. Reoxygenation started with the fast depletion of the lactate level, which fully equated with the control ones up to 6 h. Intermediates of the Krebs cycle, such as citrate and fumarate, were detected at low levels in comparison to succinate and malonate, which were elevated under the anoxic and post-anoxic treatments. The investigation of the nitrogen metabolism revealed further alteration in the metabolites. Accumulated during anoxia, Ala and GABA started to decrease, but did not reach their normoxic levels, while Gly and Pro contents increased even up to 24 h of reaeration. In contrast, the amino acids which decreased during anoxia (Asp, Gln, Leu and Thr, and slightly Val) increased during reoxygenation to the normoxic control. The levels of Asn, Cys, Orn, Ser and Met were kept low under both anoxia and reaeration. Interestingly, adenosine and guanine accumulated after the onset of reoxygenation in Arabidopsis seedlings [87].

Another set of data was obtained with rice seedlings [40]. The authors investigated the leaf metabolomes in the wild-type (M202) and nearisogenic *SUB1A* introgression line (M202(Sub1)) under sublethal submergence (72 h) and further reoxygenation (24 h). *SUB1A* controls LOQS in rice and provides more tolerance to oxygen deficiency. Again, this is a continuation of the rice metabolome profiling under oxygen limitation, as discussed in Section 2. The leaves of both the tested genotypes were characterized by the elevation in glucose and sucrose after the restoration of normal aeration. The level of glucose was changed gradually and reached a maximum substantially exceeding the normoxic one after 24 h of post-hypoxia. The sucrose content did not recover to control levels during desubmergence. Simultaneously, glucose-6-phosphate was not changed significantly in both genotypes, while sucrose-6-phosphate strived for the control level during reoxygenation, and more intensively in M202(Sub1) rice. Accumulated during submergence, lactate was downregulated upon the onset of desubmergence in plants irrespective of *SUB1A* presence. Hypoxically depleted malate content increased in both genotypes to 70–75% of the normoxic control, but was higher in M202(Sub1) rice. The succinate level temporarily accumulated in M202 and M202(Sub1), and citrate depleted, particularly in M202. Metabolome profiling revealed that desubmergence induced a significant redistribution of the amino acid pool. During oxygen limitation, the rice leaves accumulated high amounts of amino acids and reaeration provoked their significant decrease: levels of Asp, Asn, Glu, Gln, Ile, Leu, Lys and Val were reduced to such in unstressed plants, while of Ala, GABA, Met and Thr were kept over normoxic ones, particularly in the less-tolerant M202 genotype [25,40]. Reoxygenation after 1 d of submergence resulted in the almost complete recovery of the amino acid metabolism to the unstressed level in desubmerged plants [50].

Post-waterlogging for 10 days after 30 days of preceding soil hypoxia did not cause changes in the pool of sugars, and led to the accumulation of ascorbate and a number of phenolic compounds in *R. delavayi* [45].

The recovery of *L. japonicus* after 5 d of waterlogging brought about the elevation in starch, glucose and fructose levels in the roots of both wild-type and LbRNAi plants, while in the nitrogen-fixing nodules of leghemoglobin, the knockdown levels of soluble sugars were decreased [24]. Lactate was downregulated to the initial before-stress level in both genotypes. The hypoxically depleted malate level increased in the roots and nodules of the wild-type as well as the knockdown plants, while the upregulated succinate one decreased, indicating the resuming of the Krebs cycle under reoxygenation. The elevation during the waterlogging pool of amino acids (GABA, Ala, Glu, Gly, Val and Tyr) was downregulated to the initial levels, and Asp was accumulated under post-waterlogging, particularly in the roots and nodules of wild-type plants.

Unfortunately, the number of investigations focused on metabolic adaptation to reoxygenation is rather limited, and the results are still unable to provide a conclusion about the common plant metabolic profile during the transition from oxygen limitation to normal aeration. Nevertheless, some evidence indicates that the restoration of the energy supply due to the activation of photosynthesis and aerobic respiration affects the primary metabolism. The most significant metabolic changes involve the upraising of the sugar levels and the drop in the levels of anaerobically induced metabolites, including lactate, succinate and amino acids, especially GABA, Ala and Gly. Carboxylic acids showed a revival to the control level. Hence, plants which manage to survive submergence have mechanisms to restore the primary metabolism. Some results indicate that the intensity of these backwards processes collaborate with the plant tolerance towards oxygen deficiency, as well as the duration and severity of the stress factor, but further studies are needed.

## 5. Oxidative Stress Metabolomics

The production of ROS and the development of oxidative damage to different cell components are generally believed to have a major impact during reoxygenation [9,10]. Therefore, we tried to compare the metabolic profiles of plants under the conditions of post-hypoxia/-anoxia and ordinary oxidative stress, which are assumed to be associated with the overproduction of ROS that cannot be neutralized by the antioxidant system. ROS generation is well documented in plant cells due to the activity of different compartments, like chloroplasts, peroxisomes, mitochondria, the endoplasmic reticulum and plasma lemma [100,101]. It might be considered as an integral part of programmed developmental processes, like apical meristem activity, cell elongation, cell-wall formation, senescence and cell death, as well as in biochemical ones, including photosynthesis, respiration and others. Different representatives of ROS are well known to be damage-inducing agents, but also an important component of cell signaling triggering, with a wide spectrum of stress factors. The elevation in the ROS level was determined under such abiotic factors as cold, drought, wounding, intensive light, pollutants and other environmental conditions [102,103,104,105]. Special attention within the frame of our review has to be paid to ROS accumulation during oxygen deprivation and further reoxygenation. The production of ROS, especially peroxide, was determined even during the anoxic treatment [89,90,91]. Biological factors are also no less effective in the stimulation of ROS production, which would depend on the kind of factor: necrotrophs and biotrophs [106].

Alterations in the redox status affect the intensity and the direction of the plant cell metabolism. Some of the mechanisms were described with proteomics analysis as the protein redox modification [107,108]. For example, enzymes related to the carbon metabolism are among those [109,110]. Taken together, available data allow a conclusion that the post-translational redox modifications of enzymes from glycolysis and the Krebs cycle occur, and it is no wonder that intermediates of those cycles are also affected by ROS [111]. But, a very important question arises. Is it possible to distinguish metabolic events specific to oxidative stress, or will it always be mimicked by such a rich range of investigated abiotic and biotic factors? Several demanded approaches were recently developed. The treatment of plants with hormones triggered by oxidative stress in plant cells/tissues and the employment of mutants harboring the intensification/reduction of oxidative stress are in use, but they could still be considered as semidirect approaches. Moreover, a direct application of hydrogen peroxide or redox agents (for example, paraquat (methyl viologen) and cycling quinones menadione) might be considered as a key facility to clarify the ROS effect.

ROS-induced alterations of metabolomics in catalase-deficient plants [112,113,114] was reanalyzed and published in 2015 [115]. Taken together, it showed that the accumulation of a number of compounds related to the primary metabolism was revealed in *A. thaliana cat2* ROS-accumulating mutants. Among those were sugars (ribose, arabinose and rhamnose), amino acids (Arg, β-Ala, Ile, Leu, Lys, Met, Phe, Ser, Thr and Val) and organic acids (oxoglutaric, gluconic, maleic, malic and succinic acids). The intensity of such an increase was quite wide and requires further investigation of the role of peroxidases (catalase) in the regulation of ROS-induced effects on the plant metabolism.

The treatment of heterotrophic *A. thaliana* Columbia-0 cell suspension culture with 50 µM H_2_O_2_ for 1 h resulted in an intensive alteration in the protein phosphorylation level and a shift in primary metabolites (see Table S1) [116]. Starch, fructose and glucose were elevated significantly, while the levels of sucrose, glucose-6-phosphate and ribose-5-phosphate decreased. The pool of Ser and Thr increased during oxidative stress, but Pro, Gly and Met responded in the opposite way. Coming to Krebs-cycle intermediates, the decrease in citrate, 2-oxoglutarate and malate levels was discovered. Thus, in the presence of hydrogen peroxide, cells will not experience an energy lack, but the balance of amino and carboxylic acids shifted dramatically.

Another experiment demonstrated an H_2_O_2_ effect on the metabolism of the maize (*Z. mays*) cultivar BR 5011 [117]. An amount of 10 mM H_2_O_2_ solution was sprayed directly on the seedling leaves. After 2 days, the metabolite profile was detected with GS-MS. The level of glucose was slightly decreased, which was the opposite to the almost unchanged sucrose. Organic acids were also under the effect of H_2_O_2_. Lactate and succinate were accumulated to a greater extent compared to dehydroascorbate, butyrate, citrate and aconitate. The opposite decrease was detected for hexanoic, malonic and glyceric acid levels. Among the tested amino acids, the elevation was determined for Asn (the most intensive), Gly and Asp. Glu, Ser and Pro were in the list of decreased compounds.

The metabolite profile after a 20 mM H_2_O_2_ 7-day treatment was investigated in the wheat (*T. aestivum*.) cultivar of Chinese seedlings [118]. Sugars (glucose, fructose and sucrose) increased under oxidative stress. Among the organic acids, malate and citrate demonstrated elevated levels. The amino acid content was significantly increased. The upregulation was discovered for Arg, Asp, Asn, Cys, Gln, His, Phe, Pro and Val. Ala, GABA, Gly, Met, Ile, Leu, Lys, Ser and Thr levels were not changed, while Glu was downregulated.

An additional set of data about the oxidative-stress effect on the metabolome was obtained in O_3_ atmosphere [119]. Ozone fumigation (100 ppb, 10 d) was applied to thirty-day-old seedlings of soybean (*G. max*, cv. William 82). The list of characterized metabolites was quite limited. Among them, His, Asp and Arg were upregulated and citrate and 2-oxoglutarate were downregulated.

Metabolite profiling in O_3_-exposed rice leaves revealed the accumulation of a majority of amino acids (Asp, Asn, GABA, Glu, Gln, Gly, His, Ile, Leu, Met, Ser, Phe, Pro, Thr, Lys, Tyr, Trp and Val) [120]. The investigation of menadione-induced oxidative stress in rice suspension culture cells overexpressing *AtBI-1* (Bax inhibitor-1 (BI-1), a cell death suppression factor) indicated similar trends in the control and overexpressing (OX) lines, but differences in the metabolic profiling increased with time [121]. Obtained results showed that oxidative stress did not affect soluble sugar levels in all the cell lines tested. This was in a contrast with sugar phosphates (glucose-6-phosphate, fructose-6-phosphate, glucose-1-phosphate and glyceraldehyde-3-phosphate) accumulated in the OX lines, which might indicate an activation of glycolysis during oxidative stress. Pools of Krebs-cycle intermediates were not changed in both genotypes. *AtBI-1*-overexpressing rice cells demonstrated an enhancement in the content of amino acids (Arg, Asp, Asn, Glu, Gln, Met, Pro and Trp, but not GABA and Ala). Leu and NMP levels were decreased, while NTP, NAD(P)H and reduced glutathione were upregulated in transgenic lines, which was more tolerant to oxidative stress than the wild-type. The authors concluded that the overproduction of BI-1 triggers the rearrangement of the plant primary metabolism and facilitates the acclimation capacity and cell death suppression during oxidative stress.

Another set of data was obtained with the wild-type and *rcd1* (*radical-induced cell death1*) mutant of *A. thaliana* [122]. The addition of methyl viologen (paraquat) provoked the decrease in the starch and fructose levels in wild-type plants, while the mutants were very much unaffected. Sugar phosphates were downregulated in the wild-type and upregulated in *rcd1* plants. The analyzed *rcd1* mutation, which is characterized by a high tolerance to methyl viologen, had significant alterations in the sugar metabolism. The pyruvate level was decreased in the wild-type and elevated in the *rcd1* mutant upon the imposition of oxidative stress. The majority of Krebs-cycle metabolites were altered by the oxidative stress in a similar way in both genotypes. The amounts of citrate, aconitate and isocitrate were accumulated, and fumarate was depleted. Quite the opposite effect was determined for 2-oxoglutarate, succinate and malate, which were increased in the mutant and lowered in the wild-type plants. In all cases, the *rcd1* mutants contained more carboxylates than the wild-type. Some differences were detected for the amino acid metabolism. The control level was either similar or slightly exceeded in *rcd1.* Methyl viologen treatment caused the downregulation of Asp, Glu, Gly and Ser, while Gln, Ile, Leu, Lys, Pro, Phe, Tyr, Trp and Val were accumulated in the wild-type plants. The authors detected the depletion of Gly and Ser, and the elevation in Ala, Gln, His, Ile, Leu and Lys in the mutants. Surprisingly, there was no difference in Arg, Asn, Met, Thr and even GABA contents in both genotypes. The investigation assumption was that the metabolite profile of the *rcd1* mutant altered from that of the wild-type plants under light conditions. The intensification of glycolytic activity was exhibited. Under oxidative stress, the effect became more complicated. Oxidative stress markers were accumulated in the both genotypes, but the way of that accumulation indicated that oxidative stress resistance in *rcd1* mutants was very much provided by the regulation of the cell metabolism.

Recently, the effects of oxidative stress induced by ROS and ROS inducers on different groups of metabolites were comprehensively reviewed in [123]. Briefly, the obtained data indicate that H_2_O_2_ and ozone treatments induced the elevation in soluble carbohydrates, including glucose, sucrose and fructose in different plant tissues (mostly leaves) and model objects. Such an effect was also distinguished by the accumulation of oligosaccharides. Another important group of primary metabolites are the intermediates of the Krebs cycle. The addition of H_2_O_2_, menadione and methyl viologen was found to act mostly as a negative regulator of isocitrate, 2-oxoglutarate, fumarate and malate levels. This effect is in a good agreement with the results of the oxidative-dependent inactivation of Krebs-cycle enzymes. Alterations in the amino acid content were not that ambiguous. The application of ROS inducers stimulated the accumulation of Ala, Arg, Asn, Gln, His, Ile, Lys, Phe, Ser, Val, Thr, Tyr and Trp, but not of Asp, Met, Glu and GABA. For Pro and Gly, the effect was multidirectional. Thus, the reviewed data clearly indicate that the direct application of peroxide and other ROS inductors have the facility to modulate the plant cell metabolism and initiate the adaptation to this type of stressor as an independent one.

Taken together, all the considered data do not provide enough evidence to make a final supposition about the specific metabolic markers of oxidative stress, but the preliminary conclusion is that the alteration in the primary metabolism during oxidation differs from that after oxygen deprivation (reoxygenation), even if considered to be triggered with the same factors (O_2_/ROS elevation) that both activate various mechanisms.

## 6. Conclusions

**Figure 2:**
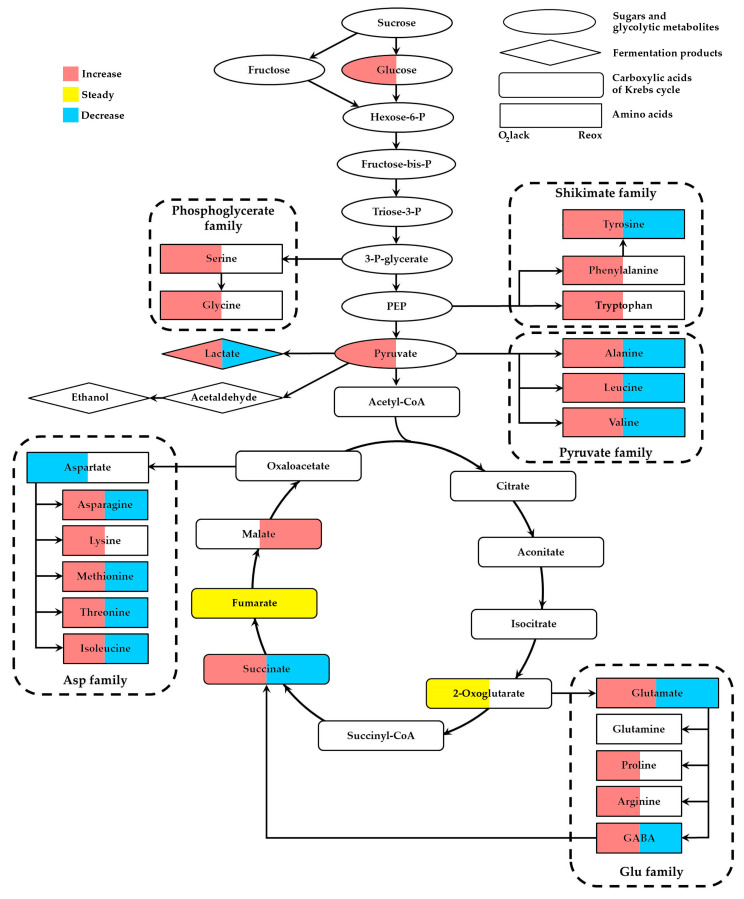
Oxygen deficiency/reoxygenation stress-specific alteration of the central metabolism in plants. Schematic representation of the major metabolic pathways of the central metabolism: glycolysis and fermentations, the Krebs cycle, the GABA shunt and the metabolism of amino acids. The scheme is based on the results of multiple studies summarized in Table S1. The frequencies of the directions in the metabolite abundance alteration after the treatments were calculated and are shown in Table S2, allowing one to choose the significantly changed metabolites (*p* < 0.1). Metabolites are indicated in different boxes corresponding to the metabolite class. Red color denotes increased abundance, yellow denotes being unchanged and blue is the decreased one. Color filling in the left part of the box designates changes due to oxygen deprivation, and in the right part, due to reoxygenation. The absence of filling reflects that the individual metabolites may display different patterns in various studies, or were not detected/measured.

**Figure 3:**
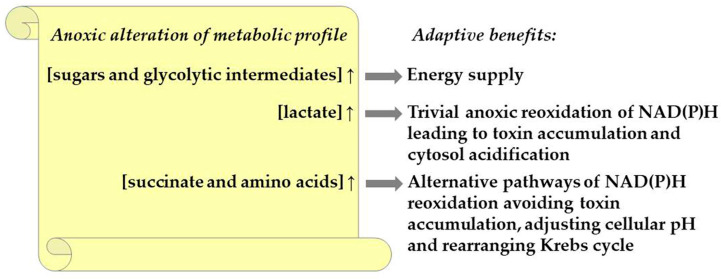
The possible importance of primary metabolism alterations in plant adaptation to oxygen deficiency.

Summing up our review, it should be noted that available metabolomics data clearly indicate significant alterations of the central metabolism in plants under hypoxia/anoxia (native or externally applied) and further reoxygenation. Integrative metabolic profiling is suitable for accelerating the discovery of the involvement of the biochemical pathways in plant responses to environmental stimuli. Unfortunately, it is rather difficult to compare the effects on the metabolism induced by the modulation of oxygen availability (native or external factors, the intensity of the oxygen lack, the duration of its application, etc.) and the trait of the studied plant for which the metabolism is under the focus (various tolerances to O_2_ deficiency, the plant organ, the stage of development, etc.). The use of different technological platforms (GC-MS, LC-MS, CE-MS and NMR) and the lack of standardization also makes it complicated to analyze the results across studies. Nevertheless, we attempted to provide a comparative analysis of the currently available metabolic profiling data under oxygen deprivation, reoxygenation and oxidative stress observed in this review (Table S1). The frequencies of the directions of metabolite abundance alteration after the treatments were calculated and are presented in Table S2. There are a number of metabolites significantly changed, with their levels forming a metabolic signature of oxygen deficiency/reoxygenation stress (Figure 2). The lack of oxygen-stimulated accumulation of glucose, pyruvate and lactate indicate the acceleration of the sugar metabolism, glycolysis and lactic fermentation, respectively. Surprisingly, among the 63 analyzed plant species, 40 were from moderately to highly tolerant to oxygen deprivation (Table S1), i.e., the results shown in Figure 2 and Table S2 concern mainly hypoxia-resistant species. Amid the Krebs-cycle metabolites, the 2-oxoglutarate and fumarate levels were unchanged, and the succinate one increased due to the inhibition of succinate dehydrogenase, aconitase and other enzymes of the cycle [28]. The amino acid metabolism was significantly altered by oxygen depletion. Amino acids related to glycolysis, including the phosphoglycerate family (Ser and Gly), shikimate family (Phe, Tyr and Trp) and pyruvate family (Ala, Leu and Val), were greatly elevated. Members of the Asp family (Asn, Lys, Met, Thr and Ile) as well as the Glu family (Glu itself, Pro, Arg and GABA) accumulated as well. Only the Asp level decreased as a result of oxygen limitation. The synthesis of amino acids during O_2_ lack provided nitrogen assimilation, osmotic and pH adjustment, the alternative regeneration of oxidized NAD(P)^+^ required for glycolysis and the production of nitrogenous compounds avoiding cytosolic acidosis (less lactate) and toxicity (less acetaldehyde and ethanol), as well as reduced carbon loss via fermentations (Figure 3) [2,8,38]. Some authors reported higher upregulation for amino acids derived from the glycolytic pathway intermediates [28], which is not surprising, since glycolysis was significantly stimulated during hypoxia. Nonetheless, the levels of Pro, GABA and Glu were also significantly elevated in anoxic plant tissues. The pathway connecting glycolysis, the truncated Krebs cycle and the amino acid metabolism is in the details discussed in [23,28]. This pathway supposes pyruvate conversion to Ala by transferring the amino group from Glu with 2-oxoglutarate synthesis. Cytosolic and mitochondrial alanine aminotransferases were upregulated by oxygen lack in different species [8,28,38,99]. The 2-oxoglutarate can be transferred to the mitochondria and be transformed into succinate by 2-oxoglutarate dehydrogenase and succinate CoA ligase, producing ATP [28,38]. The NAD^+^ required for this reaction is regenerated by malate dehydrogenase, catalyzing the reduction of oxaloacetate to malate. Oxaloacetate, in turn, is produced by aspartate aminotransferase [28,38]. To maintain this pathway, the pools of Asp and Gln would be depleted (Figure 2). Malate is either decarboxylated by the malic enzyme to form pyruvate, or reacts via fumarate to succinate in the reversed dicarboxylic part of the Krebs cycle [28]. Glu can be obtained in the GS–GOGAT cycle, which utilizes NADH and 2-oxoglutarate, but the cycle energy cost is one ATP. The GS/GOGAT cycle was shown to be activated under oxygen deficiency [99]. The excess of Glu can be decarboxylated with the production of GABA by entering a bypass branch of the Krebs cycle named the GABA shunt [2,31,38,44]. GABA is converted to succinic semialdehyde [124]. This reaction is catalyzed by GABA transaminase and coupled with the synthesis of Ala from pyruvate. Succinic semialdehyde is then converted to succinate, and then to fumarate. The oxidation of succinic semialdehyde consumes NAD^+^ and would be inhibited by oxygen shortage [44], but the conversion of GABA into succinate is reactivated upon reaeration [31,39]. Under the lack of oxygen, GABA can be metabolized to γ-hydroxybutyrate with the regeneration of NAD(P)^+^ [8]. Ala, GABA, Glu and succinate seem to be important members of the metabolic signature of oxygen deficiency connecting glycolysis with the altered Krebs cycle and allowing alternative pathways of NAD(P)H reoxidation.

It is quite interesting that alterations in the plant metabolism revealed after the modulation of the oxygen concentration in the external environment are comparable with those which occurred in native hypoxic niches, appearing in the plant organism due to developmental processes like the meristem activity or fruiting. The number of studies in this field is very limited, but even at this stage; the represented data confirm similar directions in the carbon and nitrogen metabolism adjustment, leading to the accumulation of fermentation products, succinate, fumarate and amino acids, particularly Ala, Gly and GABA.

During the plant life time, external and native hypoxia/anoxia is replaced by normal conditions, regardless of whether these plants are inhabitants of aquatic or terrestrial ecosystems. This event should restore the initial type of metabolism. Nevertheless, the available and very meager dataset indicates rather complex metabolic alterations (Figure 2). The most significant metabolic changes involve the downregulation of the levels of major anaerobically induced metabolites, including lactate, succinate and amino acids, especially members of the pyruvate family (Ala, Leu and Val), Tyr and Glu families (GABA and Glu) and the Asp family (Asn, Met, Thr and Ile). The malate level increased, reflecting the resuming of the Krebs cycle under reoxygenation. Some results indicate that the intensity of these recovery processes collaborates with the plant tolerance to hypoxia, as well as the severity and duration of the preceding oxygen deficiency, but further investigations are needed.

An additional question is raised. It concerns whether these changes are caused by the escape from hypoxia/anoxia or linked with the upcoming specific oxidative stress? A short list of investigations focused on the plant metabolism changes with ROS accumulation revealed that the main directions of metabolic alteration are rather more common with oxygen deprivation than with reoxygenation. The most significant alterations were detected in the level of soluble sugars and Krebs-cycle carboxylates [123]. Oxidative stress induced the accumulation of Gln, Leu and Val (Table S2), but not Asp, Met, Glu and GABA [123]. The most intriguing of those metabolic alterations during oxidative stress were very much similar with plant response to oxygen deprivation, but not to reoxygenation.

Emerging meanings about the metabolic signature of plant responsiveness to hypoxia/anoxia, and following reoxygenation, highlight the importance of the primary metabolism adjustment. The modification of the carbon and nitrogen pathways appears to be the most powerful. The revealed metabolic signature (Figure 2 and Figure 3) generally concerns oxygen limitation sensu lato. Currently available data do not allow us to conclude which of metabolite and/or metabolic pathways could be recognized as hypoxia- and/or anoxia-specific. It is difficult to distinguish between the metabolic profile of tolerant and intolerant plants as well. A new discipline of “omics,” called fluxomics, which quantitatively evaluates metabolic fluxes, can help to solve this problem. The advantage of this approach is based on the application of stable isotopes, as ^13^C provides a possibility to provide metabolic flux analysis [125,126]. It provides irreplaceable access to indispensable data to quantify in vivo fluxes.

Metabolomics provides a fast and rather cheap tool for metabolic phenotyping (chemotyping). The identification of a metabolic signature distinctive for plants adapted to oxygen deficiency and reoxygenation can be used to diagnose plant genotypes resistant to hypoxia, which is of certain interest for breeding activities and agrobiotechnology. Further studies are needed with a clear standardization of analytic methods, timing and intensity of anaerobic/post-anoxic exposure, etc. Very few studies pay attention to the profiling of lipophilic and secondary metabolites. It is necessary to include in future investigations a larger number of varieties, accessions and species of plants that differ in submergence tolerance.

## Data Availability

Not applicable.

## Supplementary Materials

The following supporting information can be downloaded at: https://www.mdpi.com/article/10.3390/ijms242216222/s1.
